# Transposable Elements Regulate Tail Development and Fat Deposition in Sheep Fetuses

**DOI:** 10.3390/ani15243654

**Published:** 2025-12-18

**Authors:** Qianqian Liang, Haichen Yang, Huajiao Dong, Göran Andersson, Erik Bongcam-Rudloff, Pengcheng Wan, Beibit Turganbekovich Kulatayev, Mahaba Rouzi, Min Yang, Jilong Han

**Affiliations:** 1 College of Animal Science and Technology, Shihezi University, Shihezi 832003, China15937800312@163.com (H.Y.); dhj2838132394@163.com (H.D.); 2 Department of Animal Biosciences, Swedish University of Agricultural Sciences, 75007 Uppsala, Sweden; goran.andersson@slu.se (G.A.); erik.bongcam@slu.se (E.B.-R.); 3 State Key Laboratory of Sheep Genetic Improvement and Healthy Breeding, Institute of Animal Husbandry and Veterinary Sciences, Xinjiang Academy of Agricultural and Reclamation Sciences, Shihezi 832000, China; wanpc@hotmail.com; 4 Department of Zooengineering and Biotechnology, Kazakh National Agrarian Research University, Almaty 050010, Kazakhstan; bnar68@mail.ru; 5 Xinjiang Uygur Autonomous Regional Animal Husbandry Station, Urumqi 830000, China; mhb2903@163.com

**Keywords:** transposable elements, ovine fetus, embryogenesis, RNA-seq, tail development

## Abstract

Transposable elements (TEs) play important roles in physiological processes, but their function in tail development remains poorly characterized. To explore their potential regulatory roles, we systematically analyzed the expression patterns of TEs during different fetal developmental stages of the sheep tail. Using RNA-seq data and the TEtranscripts pipeline, we identified differentially expressed TEs. Our key findings are as follows: (i) SINE/MIR, L1, and BovB elements exhibited significant stage-specific expression. (ii) The number of differentially expressed TEs was dynamic, with 2129 identified between stages E40–45 and E55–60, and 5554 between E55–60 and E70–75. (iii) These TEs were primarily located in intergenic regions, introns, and 3′UTRs, and their expression trends were highly consistent with those of adjacent differentially expressed genes. (iv) Pathway enrichment analysis revealed that TEs and their correlated genes between E40–45 and E55–60 were predominantly involved in tail elongation, while the comparison between E55–60 and E70–75 highlighted pathways related to tail fat formation. Furthermore, we identified specific candidate TEs associated with vertebra formation and fat deposition. This study provides novel insights into the role of TEs in shaping tail phenotypes and offers valuable information for sheep breeding programs.

## Simple Summary

The mechanisms underlying tail development have garnered significant attention in scientific research, particularly the factors behind structural changes and varying phenotypes. In sheep, the development of the tail vertebrae and adipose tissue is a complex process requiring precise coordination between multiple genes and signaling pathways. While transposable elements are known to be crucial in fetal development, their role in tail formation is not well understood. To investigate this, RNA sequencing was employed to analyze the tail transcriptome, including genes and transposable elements, from three stages of fetal development in lambs at gestational days 40–45 (E40–45), 55–60 (E55–60), and 70–75 (E70–75). Our findings show that transposable elements were primarily active in tail vertebra formation and elongation during the E40–45 and E55–60 stages, while significant fat deposition began at E70–75. We also identified specific transposable elements associated with these processes. This study clarifies the role of transposable elements in shaping tail phenotypes and offers valuable insights for domestic sheep breeding.

## 1. Introduction

The developmental processes of an organism reflect past evolutionary constraints and shape its future evolutionary trajectory [1,2]. The tail, an appendage with diverse functions in balance, locomotion, and communication, is a prime example of this principle. In sheep, the tail has undergone significant diversification during domestication, resulting in a variety of phenotypes classified as long fat-tailed, short fat-tailed, long thin-tailed, short thin-tailed and fat rumped [3]. This diversity, particularly the unique accumulation of adipose tissue in the tail—a trait rare among mammals–makes sheep a valuable model for studying the genetic underpinnings of complex morphological traits [4].

The genetic mechanisms governing tail development in sheep are complex, primarily involving two factors: the varying number of caudal vertebrae and the fat deposition within the tail [5]. In sheep, the fetal period begins around day 35 post-fertilization [6]. Ossification of the skeletal system commences shortly after; the vertebral column, crucial for tail structure, begins to ossify around day 46 [7,8]. Research in model organisms like mice has identified key genes regulating vertebral development, including *HES7*, *PAX1*, *Brachyury/TBXT*, and *WNT3A*, which influence vertebral number and length. Furthermore, genes such as *GDF11* and the *CDX* family are instrumental in establishing the caudal body axis [9,10,11,12]. In sheep, two linked nucleotide mutations (c.333G>C and c.334G>T) in *TBXT* have been identified that may influence the number of caudal vertebrae [13,14]. A significant breakthrough was the identification of *HOXB13*, where a deficiency leads to longer tails with more vertebrae, confirming its role in regulating tail length in mammals [15,16]. Concurrently, the deposition of tail fat is a distinct but critical process. In sheep, adipose tissue first emerges during the second trimester, with tail fat deposition becoming observable between 70 and 80 days of gestation (E70–E80), preceding the development of subcutaneous and intramuscular fat [17,18,19,20]. The advent of omics technologies has enabled researchers to conduct more comprehensive investigations into the formation of sheep tail type, leading to the identification and validation of numerous genes and biological pathways closely associated with this trait [21,22,23]. Comparisons of RNA sequence data between fat-tailed and thin-tailed sheep have implicated that *SCD*, *ESR1*, *EMR1*, and *PHYH* genes play a direct role in tail fat deposition [24]. In addition, *MTFP1*, *PDGFD* and *BMP2* have been linked to adipogenesis in sheep tail adipose tissue [20,25]. Recent single-cell transcriptomics has further revealed the involvement of *IGF2* and signaling pathways, like Hippo, TGF, and WNT, underlying sheep tail formation [26]. These findings contribute to a better understanding of the development of tail vertebrae and tail fat deposition in sheep.

Despite these advances, the role of transposable elements (TEs) in this process remains largely unexplored. TEs are repetitive DNA sequences that constitute a large portion of eukaryotic genomes and are now recognized as important players in genome plasticity, evolution and regulation, rather than “junk” DNA [27,28,29,30]. They are classified into DNA transposons and retrotransposons (including LTRs, LINEs, and SINEs) based on their mechanism of transposition [27,28]. Crucially, TEs are not silent throughout development. Specific families, such as Endogenous Retroviruses (ERVs) and Long Interspersed Nucleotide Elements (LINE-1), are highly expressed during early embryonic stages in mice, humans, and cattle, where they can act as alternative promoters and regulate the expression of developmental genes [31,32,33,34,35]. This stage-specific activation suggests a potential regulatory function in organogenesis.

Given the critical nature of the fetal period for tail development in sheep and the established role of TEs in early embryogenesis, we hypothesize that TEs are key regulators of tail formation. In this study, we leveraged high-throughput RNA sequencing to systematically analyze the expression patterns of TEs across key fetal developmental stages in the sheep tail. Our aim is to elucidate their potential regulatory relationships with both vertebral formation and fat deposition, thereby offering a novel perspective on this economically important trait.

## 2. Materials and Methods

### 2.1. Animals and Sample Collection

The Hu sheep, a prolific Chinese indigenous breed characterized by a distinctive short fat-tailed phenotype, was selected as the model for this study. This characteristic tail morphology provides a direct and relevant model for investigating the genetic mechanisms underlying caudal vertebral development and associated adipose deposition. To dynamically profile the role of transposable elements (TEs) during embryonic tail morphogenesis and adipogenesis, we analyzed three critical developmental windows (E40–45, E55–60, and E70–75 days). These stages were chosen to encompass the key periods of active tail elongation and the initiation of caudal fat deposition.

Tail tissues were harvested from twelve Hu sheep fetuses collected from a slaughterhouse in Shihezi, Xinjiang. Gestational age (40–45, 55–60, and 70–75 days) was determined by measuring crown-rump length (CRL) [36,37,38]. The samples were grouped accordingly (E40–45, E55–60, E70–75), snap-frozen in liquid nitrogen, and stored at −80 °C until RNA extraction. All animal procedures were approved by the Institutional Animal Care and Use Committee (IACUC) of Shihezi University (Protocol Number A2022-420).

### 2.2. RNA Extraction, Library Preparation, and Sequencing

Total RNA extraction was performed on tail and tail adipose tissues obtained from fetuses at different developmental stages using the RNA isolater Total RNA Extraction Reagent kit (Vazyme Biotech, Nanjing, China), strictly following the manufacturer’s recommended procedures. The quality of the RNA was determined using a NanoDrop 2000c Spectrophotometer (Thermo Fisher Scientific, Waltham, MA, USA). Then, 10 µg of total RNA from each sample was used for RNA-seq library construction. The libraries were sequenced in a single lane on an Illumina Novaseq 6000 Platform with paired-end sequencing of 150 bp read length (Novogene, Beijing, China).

### 2.3. Quality Control, Comparison, Assembly, and Quantification of Sequencing Data

The raw data from the sequencing were subjected to quality control using the fastp (v 0.13.1) software [39]. The transcriptome sequencing data of each sample after quality control were aligned to the sheep reference genome ARS-UI_Ramb_v3.0 (GCF_016772045.2) using STAR (Version: 020201) [40]. To enhance mapping sensitivity in repetitive genomic regions, including TEs, the alignment was performed with the parameters --winAnchorMultimapNmax 200 and --outFilterMultimapNmax 100. These settings allow reads to map to up to 200 potential loci per alignment anchor and retain reads aligning to a maximum of 100 genomic locations, respectively, thereby preserving multi-mapped reads for downstream TE-aware quantification. The reference genome file and corresponding annotation were retrieved from NCBI (https://ftp.ncbi.nlm.nih.gov/genomes/all/GCF/016/772/045/GCF_016772045.2_ARS-UI_Ramb_v3.0/, accessed on 20 July 2023). To visualize the global transcriptomic structure and assess the reproducibility among biological replicates across the three embryonic stages, principal component analysis (PCA) was performed on the normalized expression matrix of all detected genes and TEs. Subsequently, differentially expressed genes (DEGs) and transposable elements between adjacent developmental stages were identified using TEtranscripts (Version: 2.2.3) [41]. For each comparison (i.e., E40–45 vs. E55–60, E55–60 vs. E65–75), the earlier time point was designated as the baseline (denominator). TEtranscripts was run with the following parameters: --minread 1 to retain features with a total count of at least one across all samples, -i 10 to set the maximum number of iterations for the expectation-maximization algorithm, and --padj 0.05 to establish the significance level for calculating adjusted *p*-values (FDR). Differentially expressed features were identified by applying a dual filter of |log_2_(fold change)| ≥ 1 and an FDR-adjusted *p*-value < 0.05 to the results. Features meeting these criteria were classified as upregulated (log_2_FC ≥ 1) or downregulated (log_2_FC ≤ −1).

### 2.4. TE-Gene Co-Expression Correlation Analysis

TE-gene co-expression relationships were analyzed using sample-wise Spearman rank correlation. All included TE-gene pairs consisted of differentially expressed TEs and DEGs. Correlation coefficients (ρ) were computed based on log_2_-transformed expression values across all samples. Statistical significance was assessed using two-sided tests, with *p*-values adjusted for multiple comparisons using the Benjamini–Hochberg false discovery rate (FDR) procedure (q < 0.05). A pair was considered significantly co-expressed if it satisfied both FDR q < 0.05 and a statistical significance threshold of *p* < 0.05 prior to correction. All analyses were performed in R v4.2.0, with diagnostic plots generated using ggplot2 (v 4.0.1).

### 2.5. Functional Annotation and Pathway Enrichment Analysis of DEGs and TE-Proximal Genes

To investigate the potential biological impact of transposable element (TE) activity, we first identified TE-proximal genes, defined as all annotated protein-coding genes located within a 1 Mb window upstream or downstream of a TE locus. This set of genes was then intersected with the differentially expressed genes (DEGs) to obtain differentially expressed TE-proximal genes.

Functional annotation of the differentially expressed TE-proximal genes was performed to elucidate their biological roles. The clusterProfiler [42] and pathview [43] were used to perform Gene Ontology (GO) enrichment analysis and Kyoto Encyclopedia of Genes and Genomes (KEGG) pathway analysis on the differentially expressed TE-proximal genes. After FDR correction, a Q value less than 0.05 was considered significant.

## 3. Results

### 3.1. Tail Related Phenotypes at Different Fetal Developmental Stages

**Figure 1:**
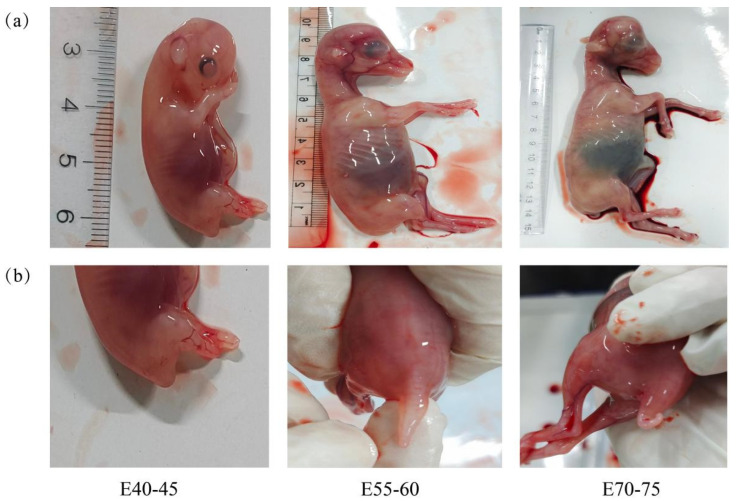
Hu sheep fetues and their tail: (**a**) Fetal Hu sheep at different developmental stages; (**b**) Tail development at different fetal stages.

Phenotypic analysis revealed distinct developmental stages: the tail underwent significant elongation from gestational days E40–45, followed by the onset of visible fat deposition at the tail base by stage E70–75 (Figure 1).

### 3.2. Distinct Developmental Clustering and Stage-Specific Transposable Element Expression

**Figure 2:**
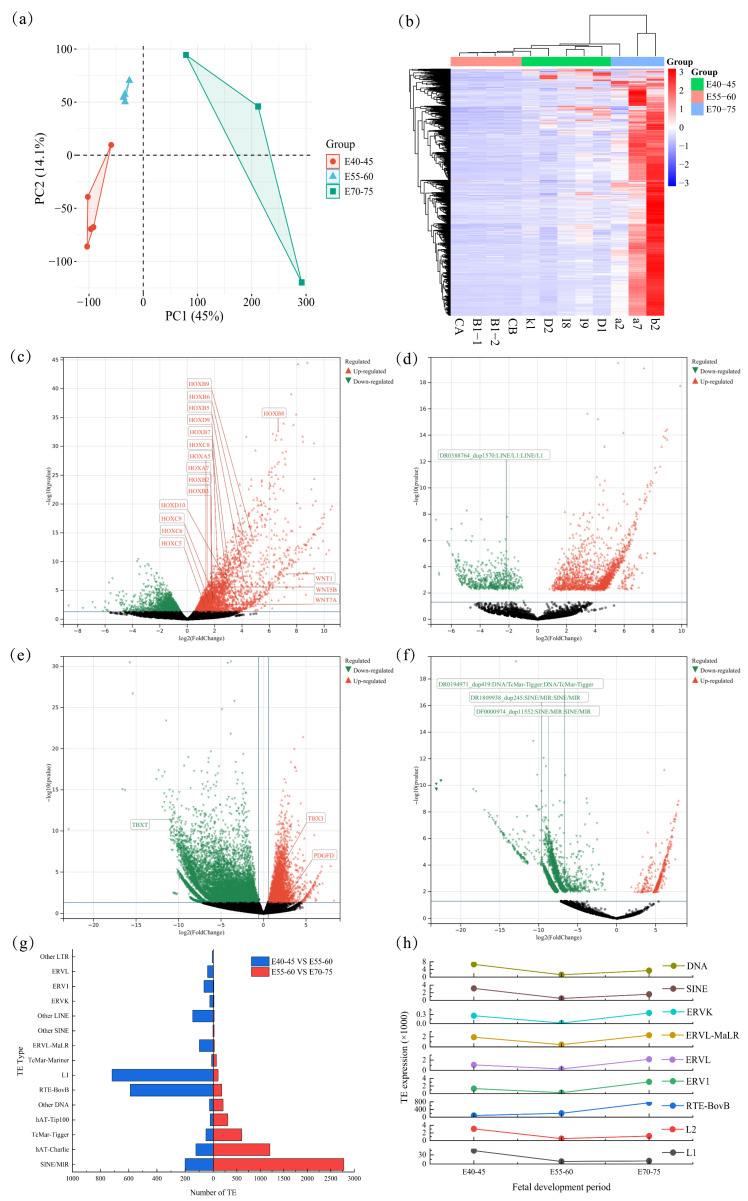
Distinct Clustering and TE Expression Signatures Across the Sheep fetal period: (**a**) PCA of Embryos at E40–45, E55–60, and E70–75; (**b**) Heatmap of Differentially Expressed TEs by Developmental Stage; (**c**) Scatter plot of DEGs between E40–45 and E55–60; (**d**) Scatter plot of TE differences between E40–45 and E55–60. In scatter plots (**c**–**f**), black dots denote genes or transposable elements (TEs) with no significant differential expression; significant ones are color-coded (up-regulated in red, down-regulated in green; significance threshold: FDR < 0.05 and |log2FC| > 1); (**e**) Scatter plot of differentially expressed genes between E55–60 and E70–75; (**f**) Scatter plot of TE differences between E55–60 and E70–75; (**g**) Statistics of different types of TEs at different developmental stages; (**h**) Expression trends of TEs during different developmental stages.

Principal component analysis (PCA) revealed clear, stage-specific clustering of the three developmental stages, with the first two principal components (PC1: 45%, PC2: 14.1%) effectively separating the samples along a developmental trajectory (Figure 2a). Samples showed clear stage-specific clustering along PC1, and the tight clustering of biological replicates within each stage confirmed high data reproducibility. This clear separation was corroborated by a heatmap of differentially expressed transposable elements (TEs), where hierarchical clustering distinctly grouped samples by their developmental stage (Figure 2b). We identified substantial changes in both gene and TE expression between consecutive stages. The transition from E40–45 to E55–60 was characterized by 2123 upregulated and 760 downregulated genes, alongside 1588 upregulated and 541 downregulated TEs (Table S1) (Figure 2c,d). A more pronounced shift occurred from E55–60 to E70–75, with 5465 upregulated and 4243 downregulated genes, and a striking reversal in TE expression: 371 TEs were upregulated, while 5183 were downregulated (Table S2) (Figure 2e,f).

The analysis of differential TEs reveals that during the E40–45 to E55–60 stages, the dynamic regulation of L1, BovB, and SINE/MIR is most pronounced. The number of L1 elements among the differential TEs is the highest, totaling 718, followed by RTE-BovB, which comprises 589 elements. SINE/MIR ranks third with 200 elements (Table S1) (Figure 2g). In the comparison between the E55–60 and E70–75 stages, the distinctions between SINE/MIR and DNA-type TEs become more apparent. Notably, the number of different SINE/MIR elements reaches 2776, while DNA/hAT Charlie follows with 1205 (Table S2) (Figure 2g). Furthermore, an analysis of the expression levels of TEs indicates that different TE types exhibit a trend of initially decreasing followed by increasing across the three periods, with all TE families showing a decline in expression levels during the E55–60 period (Figure 2h).

### 3.3. Genomic Annotation and Co-Expression of Differential TEs

**Figure 3:**
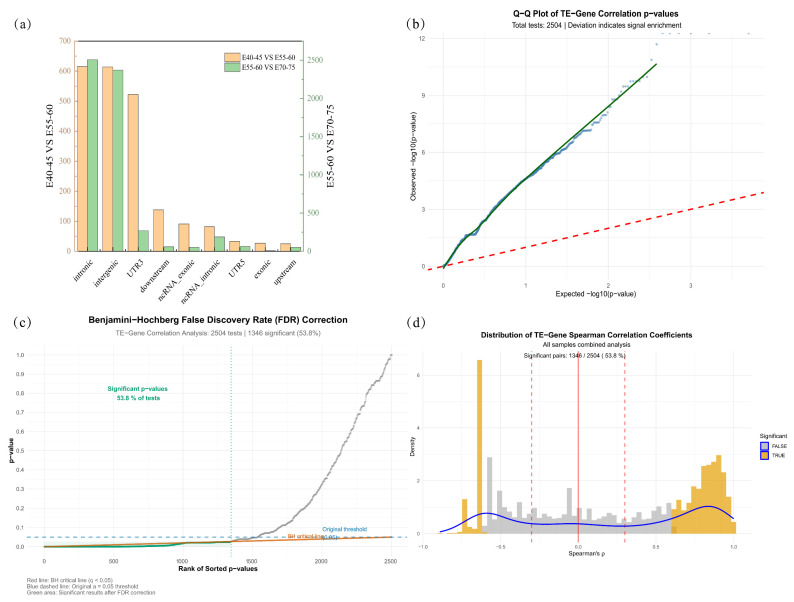
Synergistic variation in TE and host gene expression: (**a**) Genomic Distribution of Transcribed Transposable Elements; (**b**) Q-Q Plot of TE-Gene Correlation *p*-values; (**c**) Benjamini–Hochberg False Discovery Rate (FDR) Correction. The red solid line shows the BH critical threshold (q < 0.05), the blue dashed line indicates the conventional (α = 0.05) threshold, and the green shaded area highlights *p*-values that remain significant after FDR correction; (**d**) Distribution of TE-Gene Spearman Correlation Coefficients, with significant pairs (|ρ| > 0.3 and FDR q < 0.05) highlighted in orange. Vertical dashed red lines indicate the ρ = ±0.3 thresholds.

Genomic localization analysis revealed that the differentially expressed TEs were predominantly located in intergenic, intronic, and 3′ UTR genic regions (Table S3) (Figure 3a). We systematically analyzed expression correlations between differentially expressed TEs and genes. Of all tested TE-gene pairs, 53.8% (1346/2504) reached statistical significance (*p* < 0.05) before multiple testing correction, suggesting widespread regulatory interactions (Figure 3c). The Q-Q plot of *p*-values revealed a clear upward deviation of observed −log10(p) from the theoretical null distribution in the tail region (Figure 3b), confirming substantial signal enrichment rather than random noise. To control for false discoveries, we applied the Benjamini–Hochberg (BH) procedure (FDR q < 0.05). A large proportion of correlations remained significant after correction, as illustrated by the green area above the BH critical line in Figure 3c, supporting the robustness of the observed co-expression relationships. The density distribution of Spearman’s ρ for significant pairs showed a broad continuum from negative to positive correlations (Figure 3d). The peak density occurred in the positive correlation range (ρ > 0), indicating that TE expression is more frequently associated with upregulation of nearby genes. A subset of pairs exhibited negative correlations, suggesting a diverse regulatory landscape.

### 3.4. Functional Enrichment Analysis of DEGs Across Developmental Transitions

**Figure 4:**
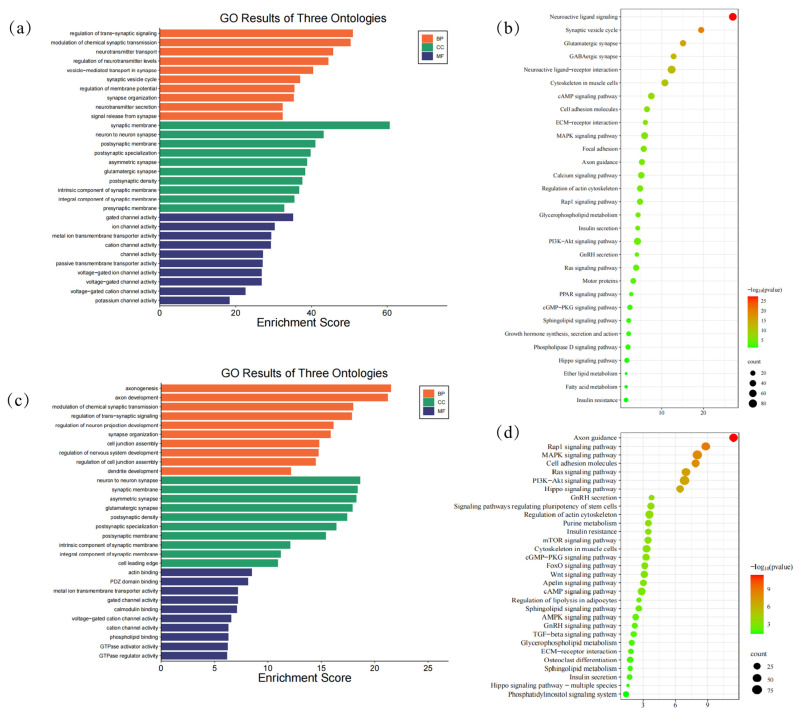
GO categories enrichment and KEGG analysis of DEGs: (**a**) GO categories enrichment analysis of DEGs between E40–45 and E55–60; (**b**) KEGG analysis of DEGs between the E40–45 and E55–60; (**c**) GO categories enrichment analysis of DEGs between E55–60 and E70–75; (**d**) KEGG analysis of DEGs between E55–60 and E70–75.

To understand the biological processes active during tail development, we performed Gene Ontology (GO) and Kyoto Encyclopedia of Genes and Genomes (KEGG) enrichment analysis on DEGs from two key transitions: E40–45 vs. E55–60 (tail elongation phase) and E55–60 vs. E70–75 (tail fat formation phase) (Table S4) (Figure 4).

Transition from E40–45 to E55–60: During the initial tail elongation phase, DEGs were significantly enriched for terms related to intercellular communication and patterning. Biological processes were dominated by the regulation of trans-synaptic signaling, chemical synaptic transmission, and neurotransmitter transport (Q-value < 0.05) (Figure 4). In cell components (CC), we identified 188 enriched terms (Q-value < 0.05), which primarily represent the synaptic membrane and neuron-to-neuron synapse. Regarding molecular function (MF), we observed enrichment of 231 terms (Q-value < 0.05) (Figure 4a). KEGG pathway analysis further supported this, highlighting key signaling pathways such as Neuroactive ligand-receptor interaction, cAMP signaling, MAPK signaling, and Axon guidance (Figure 4b). This collective profile suggests a critical role for neuronal-like signaling pathways in the early morphogenesis and patterning of the tail.

Transition from E55–60 to E70–75: In contrast, the later transition period showed a distinct functional shift. DEGs were enriched for biological processes such as fetal organ development and cell fate commitment, indicating a move from initial patterning to tissue differentiation and growth. In the category of cellular components (CC), 149 terms were significantly enriched (Q-value < 0.05), typically representing complexes such as axonemal dynein and transcription regulators. Regarding molecular functions (MF), 202 terms were identified as enriched (Q-value < 0.05) (Figure 4c). This was accompanied by the enrichment of potent, broad-acting signaling pathways including the Ras, PI3K-Akt, and MAPK signaling pathways (Figure 4d). The activation of these pathways is consistent with the onset of adipogenesis and the substantial tissue remodeling occurring at this stage.

### 3.5. Functional Enrichment of Transposable Element-Adjacent Genes

To determine if differentially expressed TEs are associated with specific biological processes, we identified their adjacent genes and performed GO and KEGG enrichment analysis (Table S5). Strikingly, the enriched terms for TE-adjacent genes closely mirrored those of the broader DEGs sets, reinforcing the key pathways active during each developmental transition.

#### 3.5.1. TE-Associated Genes in the Tail Elongation Phase (E40–45 vs. E55–60)

**Figure 5:**
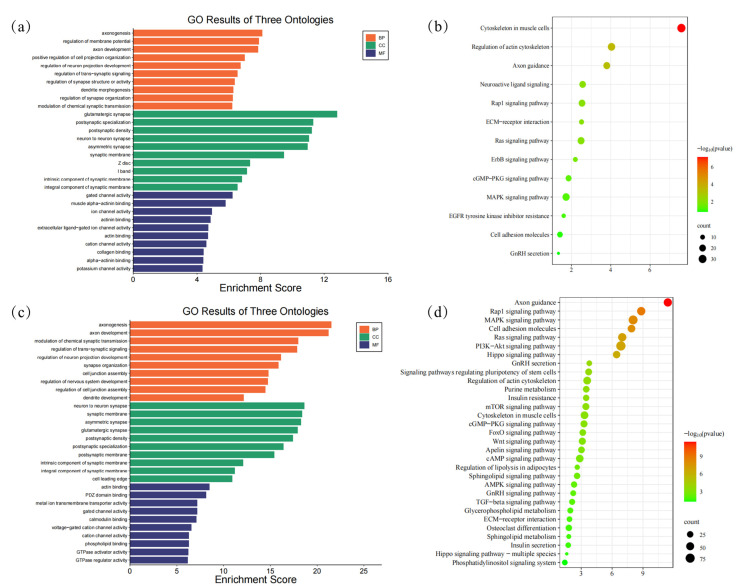
GO categories enrichment and KEGG analysis of genes related to TE. (**a**) GO categories enrichment analysis between E40–45 and E55–60; (**b**) KEGG analysis between the E40–45 and E55–60; (**c**) GO categories enrichment analysis between E55–60 and E70–75; (**d**) KEGG analysis between E55–60 and E70–75.

During the tail elongation phase, TE-associated genes were significantly enriched for processes and pathways critical for morphogenesis. TE-related genes revealed 506 enriched terms primarily encompassing axonogenesis, regulation of membrane potential, and axon development in biological processes (BP) (Q-value < 0.05). In terms of cellular components (CC), 133 enriched terms (Q-value < 0.05) were identified, with a focus on glutamatergic synapses and postsynaptic specialization. Regarding molecular function (MF), 111 enriched terms (Q-value < 0.05) were noted, including gated channel activity, muscle alpha-actin binding, and ion channel activity (Figure 5a). By integrating TE-related genes into the KEGG pathway library, we identified a total of 44 significantly enriched pathways (Q-value < 0.05), primarily including axon guidance, the Rap1 signaling pathway, the cGMP-PKG signaling pathway, and the MAPK signaling pathway (Figure 5b).

#### 3.5.2. TE-Associated Genes in the Tail Fat Formation Phase (E55–60 vs. E70–75)

In the tail fat formation phase, the analysis of TE-associated genes revealed a pronounced shift towards pathways governing growth and differentiation. We observed 1339 enriched terms related to biological processes (BP) (Q-value < 0.05), predominantly encompassing axonogenesis, axon development, and modulation of chemical synaptic transmission. In the category of cellular components (CC), 175 terms were enriched (Q-value < 0.05), primarily focusing on neuron-to-neuron synapses, synaptic membranes, and other relevant terms. Regarding molecular function (MF), 207 terms were significantly enriched (Q-value < 0.05), including actin binding, PDZ domain binding, and metal ion membrane transporter activity, among others (Figure 5c). A total of 118 significantly enriched pathways (Q-value < 0.05) were identified, with notable enrichment in axon guidance, the Rap1 signaling pathway, the MAPK signaling pathway, cell adhesion molecules, the Ras signaling pathway, the PI3K-Akt signaling pathway, and the Hippo signaling pathway (Figure 5d).

### 3.6. Identification of Candidate Transposable Elements

By integrating our transcriptomic data, we identified specific TEs whose expression was correlated with key pathways for tail development. These candidate TEs, presented in Table 1, are strong candidates for regulating tail elongation and fat deposition in sheep.

## 4. Discussion

The development of the sheep tail is a complex morphogenetic process, yet the regulatory role of TEs has remained largely unexplored. Our study provides the first comprehensive transcriptomic data providing evidence that TEs are dynamically expressed and exhibit stage-specific functions during fetal tail development. We demonstrate that the tail elongation phase (E40–60) and the fat deposition phase (E70–75) are characterized by distinct transcriptional landscapes, both for genes and TEs. Crucially, by identifying candidate TEs and showing their co-expression and genomic proximity to key developmental genes, we propose that TEs are integral components of the regulatory network orchestrating tail phenotype. The mechanisms for these effects are likely to be complex and involve both chromatin structure effects and transcriptional regulatory mechanisms.

### 4.1. TEs Are Linked to Stage-Specific Signaling Pathways

TEs play a key role in shaping genome architecture, controlling gene expression, and driving phenotypic diversity, making them crucial for evolution and domestication processes. Some TEs, in particular LTRs, can serve as *cis*-regulatory elements and therefore have the potential to drive cell-type-specific gene expression, and TEs can also act to regulate the expression of nearby genes. Our enrichment analyses directly connect TE activity to the core signaling pathways driving each developmental phase. During the tail elongation stage (E40–60), TE-associated genes were significantly enriched in pathways critical for morphogenesis, including axon guidance and MAPK signaling. While axon guidance is essential for neural development, its components are repurposed to direct cell migration and tissue patterning in diverse contexts, including the skeletal system [44,45,46,47,48,49,50,51,52,53,54,55,56]. Concurrently, the MAPK pathway is a well-established regulator of osteoblast differentiation and bone formation [57,58]. In addition, the Wnt pathway is a master regulator of vertebrate tail development and axial extension [59], and Wnt3A expression is consistently linked to tail elongation in mice [60]. *TBXT* has been consistently expressed in the same region during Wnt3 activity, and genetic studies of *TBXT* and *Wnt3A* mutant embryos indicate their critical role in axis elongation [61]. Zhang et al. [62] demonstrated that Wnt5A and Wnt11 are involved in the regulation of hippocampal caudal mesoderm and caudal mesoderm development, respectively. Furthermore, the prolonged expression of Wnt8A may facilitate the formation of the caudal axis in the hippocampus, thereby promoting the development of the cauda equina. Concurrently, Wnt ligands modulate the expression of other genes that contribute to various stages of animal development, from axoid formation to tissue formation [63]. Therefore, these effects strongly suggest that TE-associated regulation is pivotal for caudal vertebrae development. The stage-specific expression of TEs like SINE/MIR, L1, and BovB during this period therefore suggests a role in fine-tuning the expression of genes within these core pathways to coordinate caudal vertebrae development. This was followed by a profound transcriptional shift at E70–75, where TE-adjacent genes became enriched in pathways governing lipid metabolism and tissue growth. The Rap1, MAPK, Ras, PI3K-Akt and the Hippo signaling pathway were enriched for the DEGs and TEs, while the Hippo pathway centrally controls the balance between osteogenic and adipogenic differentiation in mesenchymal stem cells [64,65,66,67,68,69,70,71]. The enrichment of TEs in these pathways during the late stage suggests they contribute to initiating the adipogenic program, steering developmental resources toward fat deposition.

### 4.2. Candidate TEs Are Positioned to Regulate Key Phenotypic Genes

Vertebrate development is a dynamic spatiotemporal process that involves the division of the vertebrate body into two primary components: the precursor and the afterbody. During early embryogenesis, dynamic changes in RNA expression from TEs are associated with key developmental progressions. To move from correlation to causation, we identified specific candidate TEs located near genes with established roles in tail morphology. This provides a direct, mechanistic link between TE activity and phenotype. For instance, we identified TEs near the *TBXT* gene, a gene that was initially identified in mice [72], encodes a protein that serves as a developmental transcription factor that regulates both tail number and tail length across various mammals, including sheep [13,14]. Numerous subsequent studies have demonstrated that the *TBXT* gene is significantly present in the differential gene region distinguishing long-tailed from short-tailed sheep, thereby reinforcing its potential role in regulating tail number traits in this species [14]. It is a master regulator of tail length and vertebrae number that operates through the Wnt pathway. We also found candidate TEs associated with the *BMP2/VRTN* locus; BMP2 protein is crucial for osteogenic differentiation and tail regeneration [73,74,75,76]. VRTN is a key transcriptional regulator of BMP2 gene expression and a known determinant of vertebrae number in sheep [77,78,79,80]. Furthermore, during the fat deposition phase, we identified TEs near *PDGFD* and *SLC27A5*, genes with demonstrated roles in ovine tail fat deposition [81,82] and cellular lipid metabolism [83], respectively. The co-expression and genomic proximity of these TEs to such pivotal genes strongly suggest they act to modulate the gene regulatory networks governing tail formation.

### 4.3. A Proposed Cis-Regulatory Mechanism for TEs in Tail Development

As one of their important functional impacts on gene function and genome evolution, TEs participate in regulating the expression of nearby genes and even genes located at substantial distances at the transcriptional level. At the post-transcriptional level, TEs may regulate the expression of nearby genes, and in some cases, even genes located at considerable genomic distances. This regulation could be achieved through their potential role as cis-regulatory elements, including acting as alternative promoters, enhancers, or boundary elements. During early embryogenesis, dynamic changes in RNA expression from TEs are associated with key developmental progressions. The genomic location of these candidate TEs provides a plausible mechanism for their action. Our annotation revealed that differentially expressed TEs were predominantly located in non-coding regions, such as intergenic regions, promoters, enhancers, introns, and 3′UTRs. Moreover, our results demonstrate widespread and robust co-expression relationships between differentially expressed TEs and genes, with a predominant trend toward positive correlations. This suggests that TEs may frequently function as positive regulatory elements within transcriptional regulatory networks. This positioning is consistent with a role in *cis*-regulation. For example, intronic TEs may influence mRNA splicing, poly-adenylation or mRNA stability, while intergenic TEs may act as enhancers or chromatin modulators for distant genes. This model is strongly supported by studies showing that elements like L1 can function as a “gene regulation toolbox,” providing stage-specific enhancer activity that is crucial for orchestrating developmental processes [84]. The dynamic stage-specific expression of TEs in our study aligns perfectly with this paradigm, suggesting they contribute essential regulatory inputs to the precise spatiotemporal control of tail development. By acting as transient, modular regulatory elements, TEs can introduce the evolutionary flexibility necessary to generate the diverse tail phenotypes observed in domestic sheep.

## 5. Conclusions

Although differentially expressed TEs were predominantly annotated in intergenic, intronic, and 3′UTR regions, we identified a broad and robust co-expression relationship between these TEs and their adjacent genes. This correlation strongly suggests a potential cis-regulatory role for these TEs, providing a plausible mechanism for their influence on gene networks. Furthermore, we found that SINE/MIR, L1, and BovB families coordinate vertebral elongation during the early fetal stages (E40–E60) and subsequently drive fat deposition in later stages (E70–E75) through the Rap1, MAPK, Ras, PI3K-Akt, and Hippo signaling pathways. The discovery of candidate TEs co-expressed with and located near critical developmental genes provides a mechanistic model for their action. These findings transform our understanding of tail phenotype formation, positioning TEs as central players and opening new avenues for genetic improvement in livestock.

## Figures and Tables

**Table 1:** Information on Candidate TEs related to fetus period tail development.

TEs	Type	Insert Type	Chromosome	Start	End	Related Genes
DR0194971_dup419	DNA/TcMar-Tigger	intronic	NC_056068.1	3,978,143	3,978,830	PDGFD
DR1810544_dup1125	SINE/MIR	intergenic	NC_056061.1	88,869,734	88,869,896	TBXT
DF0000026_dup1473	DNA/hAT-Charlie	intergenic	NC_056070.1	60,187,989	60,188,100	TBX3
DR0195135_dup5673	SINE/MIR	intergenic	NC_056070.1	60,266,809	60,267,001	TBX3
DR0768748_dup3351	SINE/MIR	intergenic	NC_056070.1	60,292,019	60,292,044	TBX3
DF0000224_dup154	DNA/TcMar-Tc2	intronic	NC_056055.1	161,675,520	161,676,122	ACVR2A
DR0082726_dup584	SINE/MIR	intergenic	NC_056055.1	162,147,321	162,147,536	ACVR2A
DR0195135_dup3959	SINE/MIR	intronic	NC_056062.1	85,233,981	85,234,101	RUNX1T1
DR1068640_dup4617	SINE/MIR	intergenic	NC_056060.1	83,725,599	83,725,862	VRTN
DR0082601_dup3290	SINE/MIR	intronic	NC_056067.1	66,387,303	66,387,456	SLC27A5

## Data Availability

The data presented in this study are available on request from the corresponding author. The RNA-seq data generated in this study have been deposited in the Genome Sequence Archive (GSA) at the China National Center for Bioinformation (CNCB) under the accession number RJCA052882.

## Supplementary Materials

The following supporting information can be downloaded at: https://www.mdpi.com/article/10.3390/ani15243654/s1, Table S1. DEGs and differential TEs in E40–45 vs. E55–60; Table S2. DEGs and differences TEs in E55–60 vs. E70–75; Table S3. Genomic Distribution of TEs; Table S4. Significantly enriched GO terms and KEGG pathways of DEGs; Table S5. Significantly enriched GO terms and KEGG pathways of TEs.
